# Increased Secondary Attack Rates among the Household Contacts of Patients with the Omicron Variant of the Coronavirus Disease 2019 in Japan

**DOI:** 10.3390/ijerph19138068

**Published:** 2022-06-30

**Authors:** Tsuyoshi Ogata, Hideo Tanaka, Emiko Tanaka, Natsumi Osaki, Etsuko Noguchi, Yukino Osaki, Ayane Tono, Koji Wada

**Affiliations:** 1 Itako Public Health Center of Ibaraki Prefectural Government, Itako 311-2422, Japan; em.tanaka@pref.ibaraki.jp (E.T.); n.oosaki@pref.ibaraki.lg.jp (N.O.); e.noguchi@pref.ibaraki.lg.jp (E.N.); yu-oosaki@pref.ibaraki.lg.jp (Y.O.); a.touno@pref.ibaraki.lg.jp (A.T.); 2 Public Health Center of Neyagawa City Government, Neyagawa 572-0838, Japan; tanaka.hideo@city.neyagawa.osaka.jp; 3 Department of Public Health, Faculty of Medicine, International University of Health and Welfare, Tokyo 107-8402, Japan; kwada@iuhw.ac.jp

**Keywords:** COVID-19, Omicron variant, SARS-CoV-2, household transmission, secondary attack rate, vaccine effectiveness, sex, Delta variant, Japan

## Abstract

This study investigated the household secondary attack rate (HSAR) of patients with coronavirus disease (COVID-19) during the omicron variant-dominant period. The HSAR of COVID-19 cases during the omicron variant-dominant period (4–20 January 2022) was calculated and compared with the delta variant-dominant period (20 August to 7 November 2021) in Itako, Japan. In Itako, all 47 and 119 samples tested during the omicron and delta variant-dominant periods were negative and positive, respectively, for the *L452R* mutation. We used a generalized estimating equation regression model. The HSAR was 31.8% (95% confidence interval (CI) 27.7–36.2) for 456 household contacts during the omicron variant-dominant period; it was higher than that during the delta variant-dominant period (25.2%) (adjusted risk ratio [aRR] 1.61, CI 1.13–2.28). During the omicron variant-dominant period, HSAR was lower for the household contacts of completely vaccinated index patients (27.3%) than for contacts of other index patients (41.2%) (vaccine effectiveness for infectee 0.43, 95% CI 0.16–0.62) and was significantly higher for female contacts than for male contacts (36.2% vs. 26.1%; aRR 1.29, 95% CI 1.01–1.65). The HSAR was significantly higher during the omicron variant-dominant period than the delta variant-dominant period. The vaccination of index patients might protect household contacts.

## 1. Introduction

The omicron variant (phylogenetic assignment of the global outbreak lineage designated B.1.1.529) is a lineage of the severe acute respiratory syndrome coronavirus 2 (SARS-CoV-2) that causes the coronavirus disease (COVID-19). Omicron was first reported to the World Health Organization in South Africa on 24 November 2021 and was classified as a variant of concern (VOC) on 26 November 2021 [1]. As of 25 January 2022, among the 372,680 sequences uploaded to Global Initiative on Sharing All Influenza Data from specimens collected in the preceding 30 days, 89.1% were omicron [2]. Under the assumption of unchanged generation time, the estimated pooled mean transmission advantage of the omicron variant over that of the delta variant was 75% [3].

In Japan, the fifth wave of COVID-19 was delta variant-dominant and peaked in August 2021, and the sixth wave is omicron variant-dominant and ongoing as of June 2022 [4,5]. Patients infected with the omicron variant through the transmission in Japan were first confirmed in the latter half of December 2021 [6].

The household secondary attack rate (HSAR) characterizes virus transmissibility [7]. Studying the transmissibility of the Omicron variant and comparing it with that of the Delta variant is useful. However, few studies have reported the HSAR of the omicron SARS-CoV-2 variant [8,9,10,11,12]. The effective reproduction number of omicron is greater than that of delta in several countries [13,14,15]. A previous study reported higher HSARs among omicron variant-infected cases than among delta variant-infected cases [8]. Higher transmissibility, as well as shorter generation time, could cause the replacement of delta with omicron in Japan and many other countries.

A key objective of this study was to investigate the HSAR of the omicron variant and compare it with that of the delta variant in Japan. We also tried to ascertain the risk of the demographics on omicron variant transmission. A previous study has reported that the sex of contacts was unassociated with the HSARs of the wild-type SARS-CoV-2 strain [16]. The impact of virus variants on transmission in vaccinated and unvaccinated households is also important and can yield estimates of vaccine effectiveness (VE) [12]. Studies of the VE for the omicron variant showed that the primary series COVID-19 vaccines conferred lower protection against infection than has been observed for other VOCs [17,18,19], but a meta-analysis showed no significant difference in comparing HSAR between unvaccinated and fully vaccinated contacts for omicron [12].

## 2. Materials and Methods

### 2.1. Study Design

The study used an observational study design.

### 2.2. Setting

This study was implemented in the jurisdiction of the Itako Public Health Center in Ibaraki Prefecture, Japan, with a population of approximately 265,000. Japan consists of 47 prefectures. The local government of Ibaraki Prefecture has 10 public health centers, and each public health center has its own jurisdiction.

### 2.3. Index COVID-19 Cases

The index COVID-19 patients in this study were inhabitants in the study area with confirmed SARS-CoV-2 infection between 20 August and 7 November 2021 and 4 January to 20 January 2022.

In Japan, a public health center must be notified of all COVID-19 cases according to the Infectious Diseases Control Law (No. 104 issued in 1998) [20]. The procedure of epidemiological investigation and data collection on index patients with COVID-19 was almost the same as described in previous studies [21,22]. Patients with COVID-19 with either apparent exposure to SARS-CoV-2 outside of the household or the earliest symptom-onset date were defined as the index cases in the household.

This study defined the omicron variant-dominant period as extending from 4 to 20 January 2022. In Japan, screening for the *L452R* mutation was implemented in approximately 5–10% of samples in January 2022: the *L452R* mutation was positive in the delta variant and negative in omicron. During the omicron variant-dominant period, all 47 samples in the jurisdiction of Itako Public Health Center and 773 of 818 samples (94%) in the entire Ibaraki Prefecture were negative for the *L452R* mutation [23]. Genome sequencing confirmed that the VOC of 12 samples during the omicron variant-dominant period in the jurisdiction of the Itako Public Health Center was B.1.1.529 (BA.1). The domestic numbers of VOCs with *L452R* negative confirmed by genome sequencing were 6884 for B.1.1.529 (omicron) and 80 for B.1.1.7 (alpha) between 28 December and 24 January 2022, and most of the omicron variants were subline-age BA.1 [5].

The delta variant-dominant period extends from 20 August to 7 November 2021. In the previous study, the aim was to elucidate the HSAR of the delta variant in comparison with other variants and to evaluate the risk factors among household contacts, and data of the index patients with *L452R* positive were collected as patients with the delta variant [22]. However, the proportion of vaccinated contacts was small among contacts of the patients; we excluded vaccinated contacts in the previous study. In the present study, the data collection from index patients was extended to those without *L452R* results to compare HSAR in the delta variant with that of omicron. The *L452R* mutation was negative in several samples before 19 August 2021, in Itako. During the delta variant-dominant period, the *L452R* mutation was found in all 119 samples in Itako and 1621 of 1759 samples (92%) in the entire Ibaraki Prefecture [5,23]. Between 8 November 2021 and 3 January 2022, only one patient with COVID-19 was reported to the Itako Public Health Center.

### 2.4. Participant Household Contacts and Data Collection

The participants were household contacts of index patients with COVID-19 who lived with the patient and usually slept in the same house. The procedure for contact tracing and data collection from participant household contact patients was almost the same as described in the previous studies [20,21,22]. The Itako Public Health Center undertook SARS-CoV-2 testing of all household contacts of index cases, regardless of whether they had symptoms or not, usually on the same day or within two days of the testing of the index patients. Another test was performed as a confirmatory test if a contact had a negative test result but experienced new symptom onset during the quarantine period.

The proportions of patients including both the index patients and infected household members who were isolated at home rather than in hospital or at a hotel were 87.6% during delta variant-dominant period and 94.4% during omicron variant-dominant period. If an index patient was isolated at home, usually for 10 days, we directed that household contacts should live in rooms other than the room of the index patient in the house. When contacts could not avoid entering the room of the index patient, we directed that both should wear masks and open the windows. The quarantine period for household contacts was 14 days after symptom onset but was shortened to 10 days from 14 January 2022. The number of confirmed patients with COVID-19 living in Itako was 2813 (1.1% of the population) on 20 January 2022.

Information on household contact comorbidities was not recorded. In Ibaraki, 81% of COVID-19 vaccinations were with the Pfizer/ BioNTech vaccine BNT162b2, and 19% were Moderna vaccine mRNA-1273 for the first series of vaccinations [24]. The first series of vaccinations in Ibaraki was highest from July through October 2021. The proportion of completed vaccinations increased from 10.4% at the end of June to 75.6% at the end of October, and it was 81.2% on 20 January [25]; most of the participants had been vaccinated 3–6 months earlier. Because the booster vaccination began in January 2022 in Japan, the proportion of those who had received booster vaccination was very low during the study period. Unvaccinated individuals were defined as those who had received no COVID-19 vaccination. Incomplete vaccination was defined as being vaccinated once or twice without completing 14 days after the second vaccination before sampling or symptom onset in infected contacts. Complete vaccination was defined as being vaccinated twice and completing 14 days after the second vaccination before sampling or symptom onset in infected contacts.

### 2.5. Statistical Analysis

The HSARs in the omicron variant-dominant period were calculated and compared with regard to the vaccination status and risk factors of index cases and household contacts. The HSARs for household contacts were calculated across virus variant types. Booster vaccinations were categorized under the complete vaccination status. Based on age, the study cohort was classified into three groups as follows: ≤19 years (child and adolescent), 20–59 years (adult), and ≥60 years (older adult). For the multivariate analyses, we used a generalized estimating equation for a Poisson regression model to adjust for confounding by household cluster and calculated the adjusted risk ratio (aRR) for the secondary attack rate. We stratified household contacts by the vaccination status of both household contacts and index patients and compared HSARs using the Mantel–Haenszel chi-squared test for sensitivity analysis. Statistical analyses were performed using R version 4.4-1 (R Foundation for Statistical Computing, Vienna, Austria).

## 3. Results

**Figure 1:**
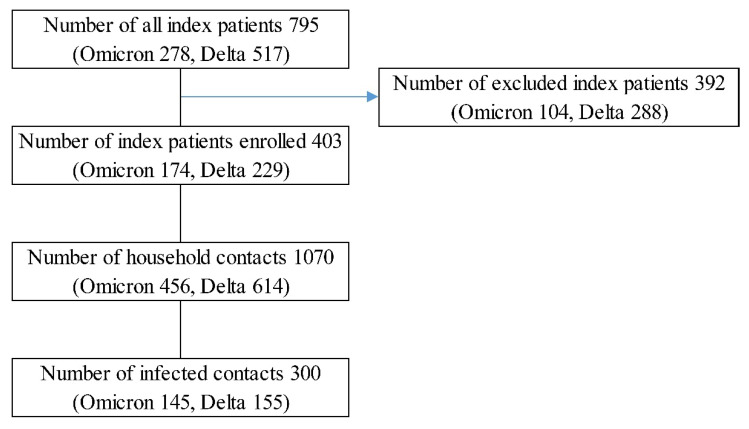
Flow chart of the enrollment of index patients and household contacts.

Table 1 and Figure 1 show the periods and enrollment. This study enrolled 1070 contacts from 403 households, and 70% of SARS-CoV-2 infections were confirmed using polymerase chain reaction tests (Table 1). The proportion of enrolled index patients in the delta variant-dominant period is smaller than that in the omicron variant-dominant period (Figure 1) because of the relatively large proportion of single households due to several outbreaks in dormitories for high school students and for foreign workers.

Table 2 shows the HSARs during the omicron and delta variant-dominant periods, and 300 of the 1070 household contacts were infected, with an HSAR of 28.0% (25.4–30.8).

The HSAR was higher for household contacts during the omicron variant-dominant period (31.8%) than during the delta variant-dominant period (25.2%; aRR 1.61, 95% CI 1.13–2.28).

Table 3 shows the prevalence of infection in 456 contacts during the omicron variant-dominant period; 145 were infected with SARS-CoV-2, and the HSAR was 31.8% (95% CI 27.7–36.2).

The HSAR was lower for completely vaccinated household contacts (28.1%) than for other contacts (41.2%). However, the aRR was not significantly lower. Among 313 contacts with complete vaccination status, only five received booster vaccinations. The HSAR was lower for the household contacts of a completely vaccinated index patient (27.3%) than for the household contacts of other index patients (41.2%; aRR 0.57, 95% CI 0.38–0.84); the VE for infectees was 0.43 (95% CI 0.16–0.62). The HSAR was higher for female (36.2%) than for male (26.1%) household contacts (aRR 1.29, 95% CI 1.01–1.65).

Table 4 shows HSARs stratified by vaccination status of household contacts and index patients. The vaccination of contacts and the vaccination of index patients are significantly related (Fisher’s exact test *p* < 0.002). When we compared HSARs by vaccination status, the *p* values using the Mantel–Haenszel chi-squared test were 0.07 for the vaccination of index COVID-19 patients and 0.02 for the vaccination of contacts.

## 4. Discussion

The HSAR of COVID-19 was 31.8% (95% CI 27.7–36.2) during the omicron variant-dominant period in Itako, Japan. The HSAR for the omicron variant was 25% in Norway [8], 49% in Spain [9], 53% in the USA [10], and 65% in Korea [11]. The HSAR in the present study was in the range of those reported previously. A meta-analysis, including studies without review, showed that the overall HSARs were 42.7% for omicron, but high heterogeneity was found [12]. HSAR for the omicron variant in the present study can provide useful data for meta-analyses in the future.

The HSAR of COVID-19 was higher during the omicron variant-dominant period than during the delta variant-dominant period by a factor of 1.6. A previous study also reported higher HSAR among omicron cases compared with delta cases [8,12]. In previous studies, the basic reproduction number of the omicron variant elicited 2.5 times higher transmissibility than that of the delta variant, and the effective reproduction number of omicron was greater than that of delta in several countries [13,14,15]. The results of the present study corroborate the findings of previous studies.

The delta variant replaced the alpha variant in England with a higher HSAR [26]. A meta-analysis showed that the overall HSAR was 29.7% for delta [12]. The omicron variant has a shorter incubation period and a shorter serial interval [27,28]. Also shorter may be the generation time, which might increase the growth rate of this variant, leading it to replace other variants. Given the increased transmissibility and low case fatality rate [5] of the omicron variant in Japan, it might be necessary to discuss the regulations on preventive interventions and coexisting with the virus in Japan.

During the omicron variant-dominant period, the HSAR was significantly lower for the household contacts of a completely vaccinated index patient than for the household contacts of unvaccinated index patients, with a VE of 41% for the infectee; this is VE against infection and not against severity. The vaccination of the index patient might protect household contacts. In preceding literature, vaccination was associated with a reduction in transmission of the alpha and the delta variants [29]. Parental vaccination confers substantial protection on unvaccinated children in the household [30]. Viable delta variant virus was detected for a notably longer duration in partially vaccinated and unvaccinated individuals compared with fully vaccinated individuals [31].

While the HSAR was lower for completely vaccinated household contacts than for other contacts, aRR was not significantly lower. However, the vaccination of contacts and the vaccination of index patients were significantly related, and the Mantel–Haenszel chi-squared test showed lower HSAR for vaccinated contacts. Therefore, the lower HSAR for the contacts of vaccinated index patients might be partially confounded by the vaccination of household contacts. A study in Japan reported that the VE in January 2022 was 49% for symptomatic patients who had received the first series of vaccinations 4–6 months earlier and was 53% for symptomatic patients who had received the first series of vaccinations more than 6 months earlier [32]. In another study, the VE for symptomatic patients aged 16–64 years in January–February 2022 were 43% and 32% among those with the first series of vaccination administered 91–180 days and >181 days ago, respectively [33].

Previous studies of the VE for the omicron variant showed lower protection of the primary series COVID-19 vaccines against symptomatic disease and infection than has been observed for other VOCs [3,17,18]. Most of the participants had been vaccinated 3–6 months earlier during the study period [25]. Therefore, the attenuation of immunity during the omicron variant-dominant period might be due to the time elapsed after vaccination completion or the increased immunological resistance of the omicron variant. A meta-analysis showed there was no significant difference in comparing HSAR between unvaccinated and fully vaccinated contacts for omicron, although HSAR for all variants combined was significantly higher for unvaccinated than for fully vaccinated contacts [12].

SARS-CoV-2 omicron-B.1.1.529 led to widespread escape from neutralizing antibody responses [34]. A study in Japan reported that two doses of the BNT162b2 mRNA vaccine did not induce sufficient neutralizing antibodies against the omicron variant compared with other variants [35]. The frequency of CD4+ T cells and CD8+ T cells reactive to peptides covering the mutated regions in the omicron variant decreased compared with the ancestral strain. Released IFN-γ was significantly reduced when cells were incubated with the peptide compared with those of the ancestral strain. However, the overall reactivity to the peptide library of the full-length protein was largely maintained [36]. SARS-CoV-2–specific T cells were detected up to 6 months after all vaccination, and no significant differences were detected between WT- and variant-specific CD4+ or CD8+ T cell responses, including omicron [37].

In the omicron variant-dominant period, the HSAR for female contacts was 36% and significantly higher than for male contacts (26%). To the best of our knowledge, no study has reported a higher HSAR for female contacts of COVID-19 cases infected with the omicron variant or other strains. Although the cause was unclear, women are often a family’s caregivers and would therefore be in close contact with the index case, and the high HSAR might also reflect a change in the ambient transmission route, such as enhancing the aerosol transmission ability of SARS-CoV-2. During the omicron variant-dominant period, home isolation has been common in Japan, and infection precaution measures were not necessarily complete. During home isolation, caregivers should take strict precaution measures in caregiving to patients.

This study had several limitations. First, the index cases could have been misclassified as secondary cases. Second, information might be biased due to the influence of the heavy burden caused by the increase in the number of patients with COVID-19 identified on contact tracing. Lastly, the omicron and delta variants were not confirmed by genome sequencing but were classified by the dominant VOC period, which was determined by detecting the *L452R* mutation. However, the *L452R* mutation test results coincided with dominant variants across >90% of samples in Ibaraki Prefecture during both periods, and genome sequencing had a coincidence between the mutation and the detection of the variant in Japan [4,5].

The cumulative incidence of COVID-19 cases was 1% during the study period. A study in February 2022 in Japan showed that 77% of reported COVID-19 patients are N antibody-positive, and 60% of participants with N antibody-positive had a history of being diagnosed as COVID-19 [38]. Further studies evaluating VE for both infector and infectee and transmission dynamics including the generation time of the omicron variant might be necessary.

## 5. Conclusions

During the omicron variant-dominant period, the HSAR was 31.8%, significantly higher than during the delta variant-dominant period. The HSAR for the omicron variant in the present study can provide useful data for meta-analyses in the future. The HSAR was significantly lower for the household contacts of completely vaccinated index patients than for the household contacts of other index patients, with a VE of 43% for infectee during the omicron variant-dominant period. Vaccination of index patient might protect household contacts, and further studies evaluating VE for both infector and infectee on omicron variant might be necessary. During the omicron variant-dominant period, the HSAR for female household contacts was 36%, which was significantly higher than that for male household contacts (26%).

## Figures and Tables

**Table 1:** Study period, enrollment of index cases, tests for case confirmation, and the number of household contacts.

	Dominant Variant during the Outbreak		Total
	Omicron	Delta	
Date	4 January to 20 January 2022	21 August to 7 November 2021	
Pandemic wave in Japan	6th	5th	
No. of all index patients	278	517	795
Number of index patients enrolled	174	229	403
Proportion of index patients enrolled	62.6%	44.3%	
Test for confirmation of index patients			
Polymerase chain reaction	112	169	281
Antigen test	56	52	108
Loop-mediated isothermal amplification test	1	7	8
Nicking enzyme amplification reaction	1	0	1
Unknown	4	1	5
Number of household contacts	456	614	1070

**Table 2:** Secondary attack rates among household contacts of index patients with COVID-19 by potential risk factors.

Variables	Household Contacts	Infected Contacts	Secondary Attack Rate	Multivariate Analysis
			% (95% CI)	aRR (95% CI)
	*N* = 456	*N* = 145	28.0% (25.4–30.8)	
Dominant viral type during the period				
Delta	614	155	25.2% (22.0–28.8)	1
Omicron	456	145	31.8% (27.7–36.2)	1.61 (1.13–2.28)
Risk factors in household contacts				
Vaccination				
0–1*	602	193	32.1% (28.5–35.9)	1
2†–3	468	107	22.9% (19.3–26.9)	0.86 (0.69–1.07)
Sex				
Male	490	121	24.7% (21.1–28.7)	1
Female	580	1*79	30.9% (27.2–34.7)	1.26 (1.07–1.48)
Age, years				
≤19	330	123	37.3% (32.2–42.6)	1.46 (1.18–1.81)
20–59	572	135	23.6% (20.3–27.3)	1
≥60	168	42	25.0% (19.1–32.1)	1.13 (0.84–1.53)
Relationship to index patient				
Spouse	155	53	34.2% (27.2–42.0)	1.49 (1.12–1.97)
Other	915	247	31.8% (24.2–30.0)	1
The size of household				
2	86	25	29.1% (20.5–39.4)	1.04 (0.70–1.54)
3	204	58	28.4% (22.7–35.0)	1.10 (0.80–1.50)
≥4	780	217	27.8% (24.8–31.1)	1
Risk factors in index COVID-19 patient				
Vaccination				
0–1*	704	203	28.8% (25.6–32.3)	1
2†–3	366	97	26.5% (22.2–31.3)	0.67 (0.46–0.96)
Diagnostic delay from onset				
≤1 days	595	155	26.1% (22.7–29.7)	1
≥2 days	436	138	31.7% (27.5–36.2)	1.30 (1.00–1.68)
Asymptomatic	39	7	17.9% (8.8–33.1)	1.03 (0.44–2.4)
Sex				
Male	533	164	30.8% (27.0–34.8)	0.82 (0.63–1.07)
Female	537	136	25.3% (21.8–29.2)	1
Age, years				
≤19	394	116	29.4% (25.2–34.1)	1.10 (0.81–1.50)
20–59	612	170	27.8% (24.4–31.5)	1
≥60	64	14	21.9% (13.4–33.6)	1.01 (0.58–1.76)

All variables were included in the analysis: aRR = adjusted risk ratio; CI = confidence interval; 1*, vaccinated once or twice without completing 14 days following the second vaccination; 2†, vaccinated twice and completed 14 days after the second vaccination.

**Table 3:** Secondary attack rates among the household contacts of COVID-19 patients during the omicron variant-dominant period.

Variables	Household Contacts	Infected Contacts	Secondary Attack Rate	Multivariate Analysis
			% (95% CI)	aRR (95% CI)
	*N* = 456	*N* = 145	31.8% (27.7–36.2)	
Risk factors in household contacts				
Vaccination				
0–1*	143	57	39.9% (32.2–48.1)	1
2†–3	313	88	28.1% (23.4–33.4)	0.95 (0.68–1.32)
Sex				
Male	199	52	26.1% (20.5–32.7)	1
Female	257	93	36.2% (30.6–42.2)	1.29 (1.01–1.65)
Age, years				
≤19	133	51	38.3% (30.5–46.8)	1.23 (0.93–1.64)
20–59	267	73	27.3% (22.3–33.0)	1
≥60	56	21	37.5% (26.0–50.6)	1.18 (0.74–1.90)
Relationship to index patient				
Spouse	72	25	34.7% (24.8–46.3)	1.34 (0.93–1.93)
Other	384	120	31.3% (26.8–36.1)	1
The size of household				
2	36	8	22.2% (11.6–38.4)	0.70 (0.37–1.32)
3	86	31	36.0% (26.7–46.6)	1.25 (0.83–1.89)
≥4	334	106	31.7% (27.0–36.9)	1
Risk factors in index COVID-19 patient				
Vaccination				
0–1*	148	61	41.2% (33.6–49.3)	1
2†–3	308	84	27.3% (22.6–32.5)	0.57 (0.38–0.84)
Diagnostic delay from onset				
≤1 days	282	84	29.8% (24.8–35.4)	1
≥2 days	150	57	38.0% (30.6–46.0)	1.38 (0.97–1.97)
Asymptomatic	24	4	16.7% (6.2–36.6)	1.01 (0.32–3.3)
Sex				
Male	233	82	35.2% (29.4–41.5)	1
Female	223	63	28.3% (22.8–34.5)	0.72 (0.48–1.07)
Age, years				
≤19	208	69	33.2% (27.1–39.8)	1.03 (0.67–1.56)
20–59	215	68	31.6% (25.8–38.1)	1
≥60	33	8	24.2% (12.7–41.3)	0.92 (0.48–1.77)

All the variables were included in the analysis. aRR = adjusted risk ratio; CI = confidence interval; 1*, vaccinated once or twice without completing 14 days following the second vaccination; 2†, vaccinated twice and completed 14 days after the second vaccination.

**Table 4:** HSARs by the vaccination status of household contacts and index patients.

Vaccination of Household Contacts	Vaccination of Index COVID-19 Patients	Household Contacts	Infected Contacts	Secondary Attack Rate
0–1*	0–1*	71	33	46%
0–1*	2†–3	72	24	33%
2†–3	0–1*	77	28	36%
2†–3	2†–3	236	60	25%

1*, vaccinated once or twice without completing 14 days following the second vaccination; 2†, vaccinated twice and completed 14 days after the second vaccination.

## Data Availability

The data presented in this study are available upon reasonable request from the corresponding author. The research data are not publicly available because of the protection of personal information.
